# Ensemble-labeling of infectious disease time series to evaluate early warning systems

**DOI:** 10.1016/j.idm.2025.12.013

**Published:** 2025-12-23

**Authors:** Andreas Hicketier, Moritz Bach, Philip Oedi, Alexander Ullrich, Auss Abbood

**Affiliations:** a Robert Koch Institute, Infectious Disease Epidemiology, Seestraße 10, 13353, Berlin, Germany; b Robert Koch Institute, Centre for International Health Protection, Nordufer 20, 13353, Berlin, Germany

**Keywords:** Outbreak detection, Early warning system, COVID-19, Surveillance

## Abstract

Early warning systems (EWSs) for detecting disease outbreaks can help make informed public health decisions and organize necessary responses. During the COVID-19 pandemic, several EWSs were proposed that use covariates such as mobility or social media data for improved timeliness and precision. Evaluating these EWSs is not trivial, since we do not have the ground truth knowledge about outbreaks of COVID-19. Workarounds for missing labels are to simulate them or produce them post hoc. Simulating COVID-19 outbreaks for evaluation is not feasible with highly complex covariates such as mobility. Furthermore, existing post hoc labeling methods do not perform well on heterogeneous COVID-19 time series. To address this evaluation gap, we propose an adaptive labeling method that produces useful labels (time-indexed annotations marking outbreak-like periods) for highly heterogeneous, nonstationary COVID-19 time series. To this end, we develop a customized ensemble of labeling methods. We find that our method consistently produces useful labels for various outbreak types, such as waves and short peaks occurring at different spatial resolutions. Lastly, we use our self-produced labels to train machine learning models and compare their performance with traditional outbreak detection methods. We find that models trained with our labels outperform classical, unsupervised outbreak detection algorithms.

## Editor highlights

• New adaptive labeling method for disease time series to retrospectively identify both outbreaks and waves.
• Generated labels allow for systematic benchmarking of outbreak detection methods on real surveillance data at different spatial resolutions, from regional to national data.
• Generated labels enable training of supervised machine learning models that outperform classical unsupervised statistical outbreak detection methods.

## Graphical abstract

**Figure:**
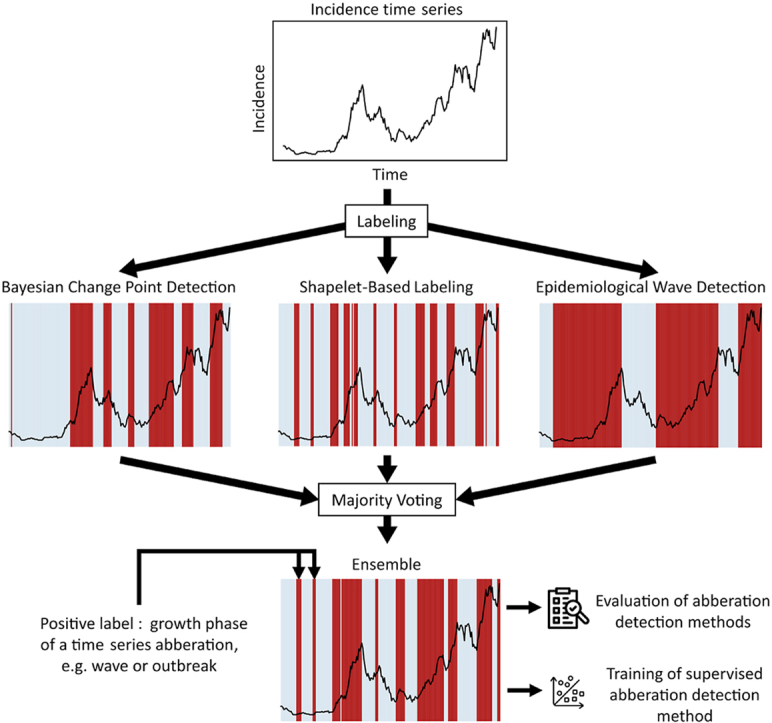

## 1 Introduction

Surveillance of infectious diseases is paramount to the timely assessment and, if necessary, the response to public health incidents (hereafter, “events,” i.e., sudden or sustained deviations from baseline consistent with potential outbreaks or clusters of cases) (Kamradt-Scott, 2019). It is desirable to have a surveillance system that detects hints for public health events (“signals”) as quickly and as reliably as possible. Early warning systems (EWSs) are crucial tools for public health experts to detect signals. EWSs are most often sophisticated compositions of algorithms and (secondary) data streams and as such, they are able to produce signals before hospitals and laboratories would pick them up through diagnostics (Kogan et al., 2021; Yabe et al., 2022; Zhang et al., 2022).

Their evaluation is not trivial, however, as ground truth data for signals are not readily available. Common evaluation strategies of outbreak detection methods make use of simulations that allow the generation of various infection scenarios with an exact knowledge of when events happened (Bédubourg & Strat, 2017). These methods are impractical for EWSs that depend on complex covariates like weather or mobility, which cannot be reasonably simulated. Alternatively, labels can be generated post hoc from lagging, gold-standard diagnostic data (e.g., confirmed case reports or laboratory positives) by identifying event-like patterns in them (Enki et al., 2016; Kogan et al., 2021; Santillana et al., 2015). A “label” here is defined as a continuous uninterrupted time-interval that annotates the original time series and is meant to represent a meaningful signal such as an outbreak or wave. These labels serve as surrogate ground truth for training and evaluation. Signals produced by an EWS can then be compared with these labels.

Existing post hoc labeling methods of gold standard data for the evaluation of EWSs do not work well for highly heterogeneous COVID-19 time series. Before COVID-19, infectious diseases would exhibit one of two salient outbreak patterns, which are namely waves (e.g., influenza) and local clusters of cases (e.g., measles) (Okhmatovskaia et al., 2022). The existing labeling methods detect these scenarios well in isolation but are not designed to detect a mix of both. During COVID-19, infectious dynamics at subnational scales were highly heterogeneous, with regional clusters co-occurring alongside travelling and seasonal waves (Jalal et al., 2024; Schilling et al., 2021; Suwono et al., 2022; Tolksdorf et al., 2022). This pattern was even more pronounced at subnational levels, i.e. the spatial scale at which EWSs are often most effective. To illustrate the difference: a new variant of concern of COVID-19 can cause a wave of infections when introduced the first time but an established variant may only cause clusters of cases, e.g., at the end of summer vacation.

Considerable effort has been invested in developing EWSs for COVID-19. In stark contrast, we are lacking the adequate methods to produce labels to evaluate EWSs for COVID-19. In this work, we introduce an adaptive, post hoc labeling method that uses an ensemble approach, which combines three established labeling strategies via majority voting to produce high-quality labels. Using German COVID-19 data, we show that the label quality of our ensemble-approach is higher compared to non-ensemble labels by visually inspecting the output, comparing it with manually curated wave-definitions for COVID-19 from public health experts, and applying descriptive statistics that evaluate the consistency of produced labels across time and space. Lastly, we use our ensemble-generated labels to train supervised learning models and show that they outperform established unsupervised outbreak detection algorithms. The respective code is published and documented to allow others to apply our method to their data.

## 2 Data

In this work, we use German data of laboratory confirmed COVID-19 cases extracted from the national infectious disease notification database Faensen et al. (2006) at the Robert Koch Institute (RKI). We use daily case counts by date of notification and aggregate by county, federal state and country. For the analyses performed in this work, we include cases that were reported between 2020 and 01–03 and 2023-10-13 to the RKI, using the database snapshot from 2023 to 10–13. The data are publicly available, see https://zenodo.org/records/8437340 for the dataset used in this work or https://github.com/robert-koch-institut/COVID-19_7-Tage-Inzidenz_in_Deutschland for the current data and a more detailed description of the data. The format of the data is described in Table 3, and examples of time series can be seen in Fig. 2.

Tolksdorf et al. (2022) performed a retrospective wave classification of the COVID-19 pandemic in Germany, assessing 15 parameters such as reported cases, test positivity, syndromic surveillance, holidays, and political measures to assign phases and identify waves of infections. These are used here as reference for our labeling method on the national level.

## 3 Methods

**Fig. 1:**
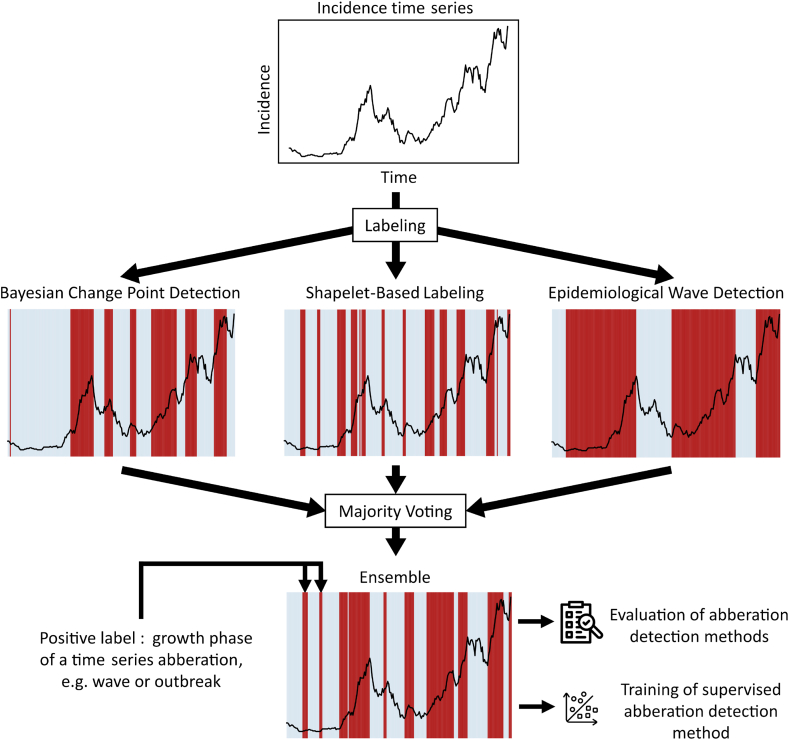
Process of the ensemble labeling method. A disease incidence time series (top) is labeled using three methods: Bayesian Change Point Detection (left, BCP), SHapelet-Based Labeling (middle, SBL) and Epidemiological Wave Detection (right, EWD). The produced labels are time-intervals shown as red areas on top of the time series. The generated labels of the three methods are then combined into one set of ensemble labels (bottom) using a majority voting scheme and several post-processing steps. The ensemble labels can then be used for evaluating existing early warning methods or to train supervised early warning methods.

The labeling approach described in this chapter generates a set of labels for each input disease incidence time series. The overall process is depicted in Fig. 1. The disease incidence time series is first labeled by three different labeling methods as described in Section 3.1. The three methods differ in their approach of detecting onset and end of the labeled time-interval but all produce the same output format, i.e. binary labels for each time point of the original time series. A majority voting scheme combines the three outputs into the final labels. Evaluating labels without a gold standard is difficult. Therefore, we describe our methods for evaluating the ensemble in Section 4. Furthermore, in section Section 4.3, we describe how the labels are used for training a supervised machine-learning based early warning method and for evaluating existing early warning methods.

### 3.1 Algorithms of the ensemble

Each of the three algorithm used in our ensemble can detect signals. These include waves as detected by the Epidemiological Wave Detection (EWD), and sudden increases of cases (outbreaks) as detected by Shapelet-Based Labeling (SBL) and Bayesian Change Point Detection (BCP). Any other algorithm can in theory be used as part of this ensemble approach if it can assign binary labels to time series at time points where event-like patterns are observed. Positively labeled time points are treated as one signal if they appear consecutively, as it is often the case with waves that span several days. The ensemble does not only depend on the selection of algorithms but also their parameters. Each algorithm has a range of settings as described in Table 1.

**Table 1:** Algorithm parameter overview.

Algorithm	Parameter	Meaning	Value
BCP	*p*_0_	Prior probability of change point	0.001
BCP	*w*_0_	Maximum number of change points to consider	1000
BCP	*cr*_ceil_	Change rate ceiling	6
BCP	*n*_days_	Window length for moving average of change rate	7
BCP	*θ*_growth_	Growth rate threshold	1
SBL	*d*_thresh_	Similarity threshold	0.8
SBL	*n*_win_	Length of shapelet in days	7
SBL	*x*_max_	Steepness of shapelet	3
EWD	*T*_sep_	Minimum temporal distance between peaks (days)	35
EWD	*P*_abs_	Absolute prominence threshold	5
EWD	*P*_rel_	Relative prominence threshold	0.01

We use the following notation for all algorithms: $X=\left\{x_{t}:t\in \left\{1,\ldots ,n\right\},x_{t}\in R\right\}$ is the time series of incidences or case numbers. $Y=\left\{y_{t}:t\in \left\{1,\ldots ,n\right\},y_{t}\in \left\{0,1\right\}\right\}$ is the time series of labels, i.e. the output of each algorithm.

#### 3.1.1 Bayesian Change Point Detection

Change point detection is a method to identify points in sequential data where the statistical properties of the data change. For outbreak detection we are interested in a change in the mean of the growth rate as this allows us to identify points where we can expect an increase or decrease in case numbers. Change point detection has also been applied to public health data by Texier et al. (2016).

We are primarily focused on the variations in the growth rate rather than the direct time series data of cases or incidences represented by *X*. Formally, let *G*_*t*_ = *X*_*t*_/*X*_*t*−7_ denote the 7-day growth rate of the COVID-19 incidence at time *t*. We use a 7-day window to account for intra-week seasonality in case reporting, as COVID-19 case reports typically exhibit weekly patterns with reduced reporting during weekends and increased reporting at the beginning of the week. Now let *U* = {*u*_*t*_: *t* ∈ {1, *…*, *n*}, *u*_*t*_ ∈ {0, 1}} denote the time series of possible change points, where *u*_*t*_ = 1 denotes a change point at time *t* + 1. We use $g_{t_{i},t_{j}}$ to denote the series of observations between change points at times *t*_*i*_ and *t*_*j*_. We are interested in *p*_*t*_, the probability of a change at time *t* + 1, as well as *μ*_*t*_ the mean of observations $g_{t_{i},t_{j}}$. Barry and Hartigan (1993) derive a Bayesian method for estimating *p*_*t*_. The bcp package by Erdman and Emerson (2007) implements a Markov chain Monte Carlo (MCMC) procedure to estimate *p*_*t*_.

We use the bcp package to obtain a time series of estimated means ${\hat{\mu }}_{t}$ representing the estimated mean growth rate within the current segment (i.e., from the most recent change point up to time *t*), and then generate a series of labels with

$$y_{t}^{\text{BCP}}=\left\{\begin{array}{ll} 1\; & \text{if }{\hat{\mu }}_{t}>\theta_{\text{growth}}\\ 0\; & \text{otherwise,} \end{array}\right)$$

where *θ*_growth_ = 1. This threshold is chosen because the 7-day growth rate *G*_*t*_ = *X*_*t*_/*X*_*t*−7_ compares current incidence to the incidence seven days prior: values above 1 indicate increasing case numbers (exponential growth), while values below 1 indicate declining trends. Since we aim to detect outbreak-like patterns characterized by growth, we label time periods where the estimated mean growth rate exceeds this natural threshold. We will refer to the algorithm as Bayesian Change Point Detection (BCP).

#### 3.1.2 Shapelet-Based Labeling

A shapelet is a characteristic subsequence that represents a specific pattern within a time series. In time series classification, shapelets are used to identify discriminative patterns: the algorithm searches for subsequences in the training data that best distinguish between different classes, and the similarity between these learned shapelets and new observations serves as a classification feature (Hills et al., 2014). Beyond classification, Srivastava et al. (2022) demonstrated that shapelets can also be used to quantify similarity between time series in the context of forecasting evaluation. We adapt this similarity-based approach for the task of time series labeling.

At its core, the algorithm assigns labels to specific time points based on the similarity of their neighborhood to a predefined shapelet. To this end, we introduce a shapelet of size *n*_win_ denoted as *S* = {*s*_*i*_: *i* ∈ {1, …, *n*_win_}}, where *n*_win_ = 2*k* for some k∈Z (i.e., *n*_win_ is even). We also introduce the neighborhood of time point *t* as $W_{t}=\left\{x_{t-n_{\text{win}}/2},\ldots ,x_{t+n_{\text{win}}/2}\right\}$ and the Pearson correlation between *S* and *W*_*t*_ as *ρ*_*t*_(*S*, *W*_*t*_). Given a similarity threshold *d*_thresh_, we can then generate a series of labels *Y*^SBL^ such that:

$$y_{t}^{\text{SBL}}=\left\{\begin{array}{ll} 1\; & \text{if }\rho_{t}\geq d_{\text{thresh}}\\ 0\; & \text{otherwise.} \end{array}\right)$$

In the context of EWSs, our primary objective is the detection of uptrends or similar emerging patterns in time series of incidences or case records. For this purpose, we use the “surge” shapelet introduced by Srivastava et al. (2022), which provides a suitable representation of such uptrends. The *i*-th element of the surge shapelet vector is defined as:

$$s_{i}=f\left(n_{\text{win}},x_{\text{max}},i\right)=2^{\frac{\left(i-1\right)x_{\text{max}}}{n_{\text{win}}}}$$

Here, the parameter *x*_max_ controls the steepness of the shapelet, offering a versatile means to shape its characteristics. We will refer to the algorithm as Shapelet-Based Labeling (SBL).

#### 3.1.3 Epidemiological Wave Detection

We apply the Epidemiological Wave Detection method developed by Harvey et al. (2023). This method identifies epidemiological waves as sustained periods of increase in case numbers that are sufficiently substantial and significant to be considered meaningful. The approach is based on identifying local extrema (maxima and minima) in the time series and applying prominence-based filtering to distinguish genuine waves from minor fluctuations.

The algorithm first identifies all local maxima and minima in the incidence time series. These extrema are then filtered based on two prominence criteria: absolute prominence *P*_abs_, which requires peaks to exceed their surrounding valleys by at least this threshold value, and relative prominence *P*_rel_, which filters peaks based on their height as a proportion of the maximum peak. Additionally, the temporal separation parameter *T*_sep_ specifies the minimum time distance (in days) required between successive peaks to be considered distinct waves, preventing the detection of multiple peaks within short time periods.

After filtering, the remaining local maxima represent wave peaks, and the minima represent wave boundaries (start or end points). We align these sets by including the first observation as the first minimum. In cases where the last extreme point is a minimum, we use the last observation of the time series as the maximum of the current wave. This results in a set of pairs of extrema *E* = {(*t*_min,*j*_, *t*_max,*j*_): *j* ∈ {1, …, *n*_waves_}, *t*_min,*j*_, *t*_max,*j*_ ∈ {1, …*n*}}, where *n*_waves_ is the number of detected waves and *j* indexes the individual waves.

Since we are most concerned with the onset of a wave, we assign a label to all time points between a minimum and the next maximum:

$$y_{t}^{\text{EWD}}=\left\{\begin{array}{ll} 1\; & \text{if }t_{\text{min},j}\leq t\leq t_{\text{max},j}\text{ for any wave }j\\ 0\; & \text{otherwise.} \end{array}\right)$$

In contrast to the original implementation by Harvey et al. (2023), we removed the population size dependency from parameter *P*_abs_ and set it to a constant value (see Table 1). By doing so we eliminate two additional parameters while still preserving good behavior. We also set *P*_rel_ to near zero, which effectively removes the last subalgorithm step. We include their code in our own package with kind agreement by the authors. We will refer to the algorithm as Epidemiological Wave Detection (EWD).

#### 3.1.4 Ensemble-labeling

Ensemble methods are a powerful class of machine learning techniques that combine the predictions of multiple individual models to create a more robust and accurate final prediction. Ensembles often exceed the performance of single methods even if the ensemble is comprised of weak models.

In the context of time series labeling, a voting mechanism is a common way to aggregate the outputs of individual models in an ensemble. For instance, when multiple models are employed to label points within a time series, the majority vote or a minimum number of votes *n*_min_ can be used to determine the final label for a particular point. If the majority of models agree or a minimum number of votes is exceeded on a specific label, that label is assigned. We use majority voting for all reported results.

### 3.2 Implementation in python package

We provide an open-source package named *epylabel* that implements the algorithms above in Python. The package can be found on GitHub and Zenodo (Hicketier et al., 2024). The package employs a pipeline approach that can be used as is or can easily be customized. For instance, to generate only the labels of the BCP algorithm, the pipeline looks like this:

bcp_labels = Pipeline([Changerate(n_days = 7, changerate_ceiling = 6), Bcp(d = 1000, p0 = 0.001, thresh = 1) ]).transform(input_data)

In the snippet, the input data is first used to calculate the change rates, then the change point detection is applied on that result. It is also possible to pass multiple data objects to one algorithm, as we do with our ensemble:

final_labels = Pipeline([Ensemble(n_min = 2)]).transform(bcp_labels, sp_labels, wf_labels).

## 4 Evaluation methods

The most common approach to evaluate post hoc labeling methods and in general aberration detection algorithms is to produce synthetic time series of case counts and to simulate events (such as outbreaks). In the absence of real labels, using labels generated by our ensemble labeling approach comes closest to a ground truth required to perform numerical evaluation of algorithm performance. This is an accepted approach when data synthesis is considered an adequate replacement for real data, which is usually the case for well-understood and consistent infection patterns. A similar option is lacking for COVID-19. To evaluate the quality of our generated labels we first do a descriptive analysis, followed by checking the spatial consistency of the labels. Lastly, we use our generated labels to train supervised outbreak detection models and compare them to traditional, unsupervised outbreak detection models on a held-out test data set.

### 4.1 Descriptive analysis

Without a simulation-based evaluation method, we fallback to visual inspections and consistency checks of labels produced by our three labeling algorithms (BCP, SBL and EWD) and their ensemble. This comes close to the screening process of an epidemiologist who will visually examine time series of infection counts for signals as part of their surveillance activities. We furthermore performed consistency checks through descriptive analyses. Specifically, we investigated the length and the number of labels produced for the different labeling approaches and spatial aggregations. We compared the label statistics to inspect whether the ensemble approach indeed yields a compromise of label properties compared to the individual labeling methods.

### 4.2 Spatial consistency

We clustered labeled time series to examine the spatial and temporal homogeneity of our self-produced labels. The assumption is that events start locally and labels for nearby counties will be better aligned temporally compared to distant counties. The cluster quality helped us assess the consistency of our ensemble approach. To this end, we apply agglomerative hierarchical clustering using complete-linkage and the Jaccard distance metric for evaluation.

### 4.3 Applications

We explore two use cases for the application of our generated labels: i) We use the labels to train supervised machine learning models and ii) we use the labels to evaluate the performance of ML models, as well as to evaluate the performance of a classical outbreak detection algorithm.

#### 4.3.1 Supervised learning outbreak detection

For the training and evaluation of supervised learning models for outbreak detection, we used time series classification models from Python's library sktime (Löning et al., 2019, 2022). We chose time series classification models because they are specifically designed to capture temporal patterns and dependencies in sequential data, making them well-suited for detecting outbreak patterns in epidemiological time series. Since time series classification is defined to produce one output label per time series, we rearrange the data into matrix form. The time series of length *n* is divided into a set of time series of length *m*, producing a (*n* − *m* + 1) × *m* matrix. *m* is set to 21 and can be thought of as a sliding window over the time series. Since we do not use padding, the first prediction we can make is for *t*_20_ of our original time series. To predict a label for day *t*_*j*_, the model has *m* days of data available (*t*_*j*−*m*+1_ to *t*_*j*_).

All performance metrics are produced using a five-fold cross validation (where the data is split into five subsets, with each subset serving once as test set while the remaining four serve as training sets) and by reporting the average performance across all folds.

We trained various models using their default hyperparameters. We report only the results for the best performing model (RotationForest) in the main text (Table 2). For a full list of results, please see the appendix (Table 4).

**Table 2:** Performance comparison of outbreak detection algorithms. A supervised learning model (rotation forest) was trained on our ensemble labels and evaluated using a five-fold cross-validation. For comparison, an unsupervised outbreak detection algorithm (EARS) was used. It was evaluated on the same test folds as the rotation forest. We used machine learning metrics for evaluation, namely precision (prec.), recall, and specificity (spec.). Additionally, we used measures of timeliness: the average days until detection (DTD), the probability of detection (POD), and the POD within five days (POD_5_). Higher values relate to better performance except for DTD (indicated by arrows). The experiments were repeated for three spatial resolutions, DE is the country-level, BL the federal-level, and LK the county-level.

Region	Paradigm	Model	Prec. *↑*	Recall *↑*	Spec. *↑*	DTD *↓*	POD *↑*	POD_5_*↑*
DE	supervised	RotationForest	0.81	0.85	0.80	1.50	0.92	0.89
DE	unsupervised	EARS	0.93	0.17	0.99	4.72	0.85	0.56
BL	supervised	RotationForest	0.75	0.79	0.78	2.62	0.88	0.78
BL	unsupervised	EARS	0.87	0.16	0.98	5.52	0.82	0.68
LK	supervised	RotationForest	0.60	0.58	0.80	3.03	0.73	0.63
LK	unsupervised	EARS	0.78	0.16	0.98	4.22	0.69	0.64

#### 4.3.2 Evaluation of outbreak detection methods

We used a classical outbreak detection method as a baseline to the supervised learning model. Specifically, we used the Early Aberration Reporting System (EARS) C1 method as described in Fricker et al. (2008) and implemented by Salmon et al. (2016) in the surveillance package. EARS was chosen as baseline because it is a widely-used, established method in public health surveillance that operates without requiring labeled training data, making it a suitable reference for comparison with our supervised learning approach.

EARS C1 uses a sliding window of the previous seven days to calculate the mean and standard deviation for outbreak detection. While EARS C1 could produce predictions starting from day 8, we discarded predictions for the first *m* days to ensure a fair comparison with the ML models, which can only produce predictions starting from *t*_20_. EARS C1 was applied to the entire time series without cross-validation, whereas the ML models were evaluated using five-fold cross-validation as described above.

To evaluate the results, we computed precision, recall, and specificity on a per-day basis. For each day in the time series, the model produced a binary prediction (outbreak vs. non-outbreak), which we compared to the corresponding ensemble label for that day. Precision is defined as the proportion of predicted outbreak days that are true outbreak days. Recall (also known as sensitivity) is the proportion of true outbreak days that are correctly predicted, i.e. days within a labeled period for which the model also raises a signal. Specificity is the proportion of true non-outbreak days that are correctly predicted as non-outbreak days. Thus, an outbreak period contributes true positives only on those days where the model predicts an outbreak; a single positive prediction somewhere within a labeled period is not sufficient to obtain a high recall. Additionally, we reported a set of epidemiological metrics related to timeliness: (i) Days to detection, which is the number of days that have passed between the detection and the onset of an outbreak. If the model is too early, it is assigned a value of 0. (ii) Probability of detection (POD), reaches a maximal score of 1 if the model made at least one correct prediction within all outbreaks/waves in a time series and 0 if no outbreak/waves are detected. Lastly, we used a variation of POD which will treat a correct prediction within 5 days after onset also as predicted (probability of detection within 5 days (POD 5)). While precision, recall, and specificity are calculated per day, timeliness metrics are calculated per period of consecutive labels (e.g., waves).

## 5 Results

There is no gold standard dataset for COVID-19 in Germany to which generated labels could be compared to. Consequently, evaluating the generated labels, which are the result of an unsupervised learning method, is difficult. Instead, we conducted descriptive analyses of all labels and investigated whether the ensemble method produced consistent and useful labels.

**Fig. 2:**
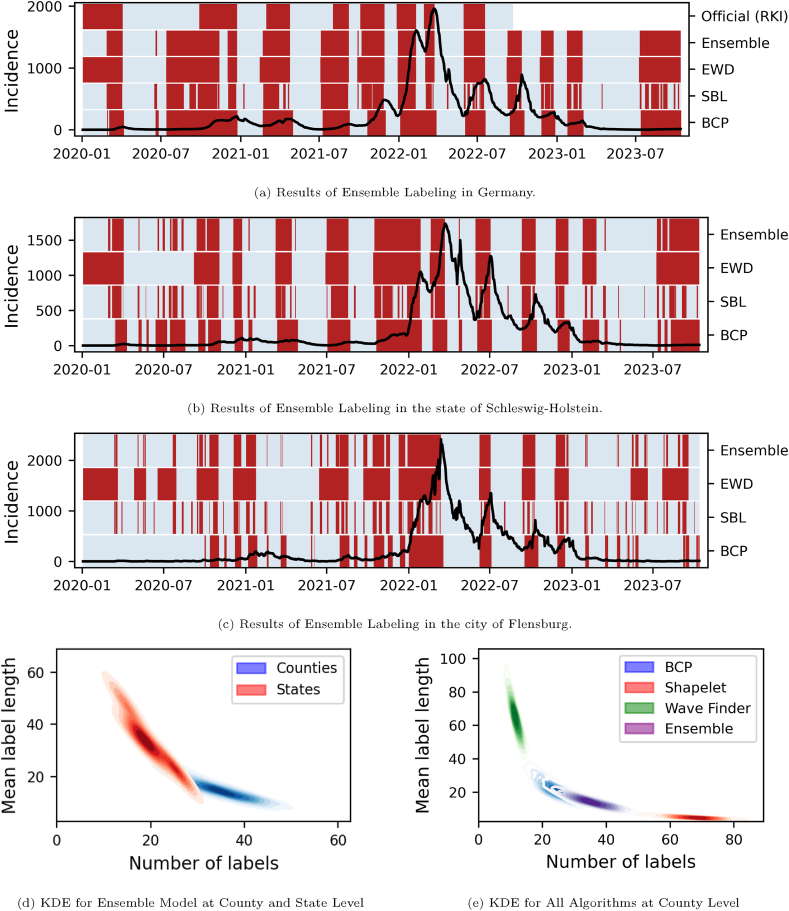
Description of ensemble labeling results. (a–c) show labeled COVID-19 7-day incidence time series on country, federal, and county level and (d,e) show descriptive statistics of the labels. Sub-figure (a) shows the German COVID-19 incidence in black with the official wave definition from the Robert Koch Institute, signal labels produced from the three base algorithms, as well as the ensemble labels. Labeled time periods are indicated by red bands, otherwise the time series is gray. Sub-figure (b) shows the COVID-19 incidence with labels from the base algorithms and the ensemble method for the German state Schleswig-Holstein and (c) for the county of Flensburg. Sub-figures (d,e) show kernel density estimate (KDE) plots to visualize the joint distribution of the number of labels (x-axis) and mean label length (y-axis). Each colored region (KDE) represents the estimated density of label characteristics across all time series within a group. (d) compares the distribution of label characteristics for ensemble labels across all German federal states (16 time series) versus all German counties (411 time series). (e) compares the distribution of label characteristics for different labeling methods (BCD, Shapelet, EDW, and their combinations) applied to all counties.

Our descriptive analyses aimed at understanding if the ensemble method indeed produced labels with properties that are comprised of those of the single methods. Fig. 2 shows the different label results on a small sample of time series and a visual representation of how the number of labels and the average label length behaves for the different labeling methods.

**Fig. 3:**
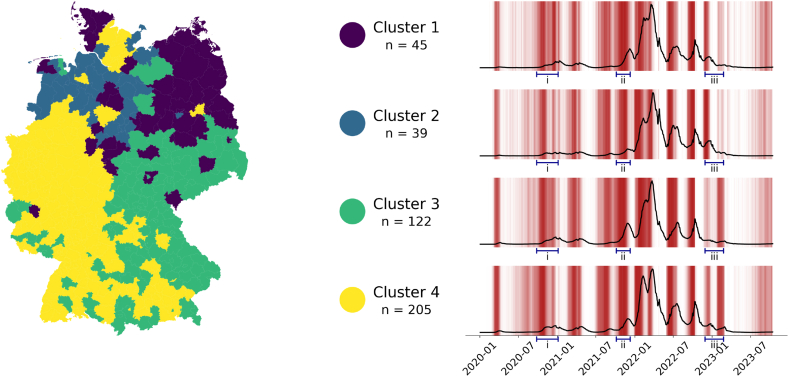
Spatial coherence of ensemble labels. Left: Map of Germany where counties are colored according to their cluster membership. Clusters are based on time series of the county-level ensemble labels; Right: Plot of the average incidence time series and average label density per cluster.

Labels were also examined on how consistent they were. Given how contagious COVID-19 is and Germany's dense population, infection patterns and therefore their labels should be correlated temporally and spatially. Fig. 3 shows our result on the spatial and temporal consistency of ensemble-generated labels. A clustering method was applied to assign labeled time series of all German counties to one out of four clusters.

Lastly, we show that labels are useful by assessing them in a supervised learning experiment. A supervised learning algorithm should be able to learn from these labels and outperform unsupervised methods. The results of our experiments using various machine learning and epidemiological performance metrics can be found in Table 2.

### 5.1 Descriptive analyses

A visual inspection of a small sample of time series shows how the ensemble method produces labels that are a compromise of the single labeling methods (Fig. 2(a–c)). SBL produced comparably many labels that were temporally short. The number and the shortness of labels increased the higher the spatial resolution (increasing from Fig. 2a–c). It is the only method that produced label lengths smaller than one week on national level and label lengths that did not extend over one month on county level. BCP, compared to SBL, produced fewer and temporally longer labels that increased in number and became shorter with increasing spatial resolution. It also had labels lasting several weeks even on county level. Lastly, the EWD produced fewer labels that all lasted several weeks on all spatial levels. These labels matched the official wave declaration of the Robert Koch Institute (Fig. 2 (a)) quite well except for the summer 2020, where EWD detected the start of a wave but was wave-free according to Robert Koch Institute.

The ensemble, which labels if two single algorithms agree to produce a label at the same time points exhibited a compromise between the single algorithms. It produces much more and shorter labels on county-level compared to EWD but is also able to produce wave-like labels on country-level as EWD.

Fig. 2 (d, e) show 2-dimensional kernel density estimates (KDEs) for the number and average length of labeled periods. In subplot (d), the density estimates on federal level and county level show that labeled periods in counties are on average shorter (10–20 days long) but that their time series have more labels (up to almost 50). The incidence time series for the smaller regions (not shown) are more erratic due to lower case numbers, which leads to a greater number of labels. On federal level, time series have on average 20 labels and label lengths of more than 50 days. The ensemble delivers reasonable results for different levels of spatial aggregation and both distributions seem well separated.

The kernel density estimate plot in Fig. 2 (e) compares number of labels and average label length of all single algorithms, combinations of two algorithms and the full ensemble calculated over all county. As seen in Fig. 2 (a), EWD produces the fewest and longest labels with less than 20 labels but with lengths of up to 100 days. On the other end of the spectrum, SBL produced by far the most labels with over 80 and has the shortest label length together with the other twosome-ensembles containing SBL of far less than 20 days.

To relate our labels to an independent wave detection framework, we additionally compare them to the recently proposed EpidemicKabu method for COVID-19 wave detection (Galvis et al., 2024). We perform a cross-comparison by applying EpidemicKabu to our German data and our ensemble method to the original EpidemicKabu time series (15 different countries), with details provided in Appendix 11.3. We find that our method works well on these different time series and observe broad agreement between the two methods.

### 5.2 Consistency

Due to the high infectivity of COVID-19, we assume that increases of cases will quickly lead to an increase of cases in neighboring counties. Similar infection dynamics of neighboring counties should lead to temporally close and similar labels. We used clustering algorithms to validate our assumption of spatial and temporal consistency of labels at the county level. These time series do not contain any incidence data or information which county is neighboring another county. Remarkably, the clusters show a clear spatial coherence, as can be seen in the clustered map of Germany in Fig. 3. The first cluster contains mainly counties in the northeast of Germany, the second contains only counties in the northwest. The third contains mainly counties in the east and south though the pattern is a bit more diffuse. The fourth cluster contains mainly areas in the west and south but, interestingly, also some of the biggest cities in the north (Berlin and Potsdam, Hamburg, Hanover, around Bremen).

These clusters intuitively make sense but they also mirror official designations like the Kleeblattkonzept (Clover Leaf Concept, Gräsner et al. (2020)) and observations from other studies from Schuppert et al. (2021) that showed that in the East of Germany, the waves in the end of 2021 differed substantially from the behavior in the other regions. Importantly, the clusters avoid being diffuse and randomly distributed all over the map, which would have falsified our hypothesis of spatial coherence. Fig. 3 also shows the average time series of each cluster as well as the proportion of counties that are labeled for each day. Differences here are harder to spot, since they are all still from the same country, but they do exist. Cluster two has a lower third and fourth wave, cluster three is missing the last wave (and accordingly the labels). To point out three examples: (i) Comparing the different clusters during the second wave of the pandemic, the second cluster shows smaller levels of incidence as well as lower label frequencies. (ii) In the period from January 2022 to the end of march 2022, there is a strong increase in incidence levels among all clusters. While the exact shape of the time series curves differs between them, cluster number four is the only one with two clearly distinct peaks. Our labels correspond well there, with two distinct label phases. (iii) Clusters 1 and 2 show a pronounced increase of incidence and predicted positive labels in November and December of 2022, while this dynamic is spread out across two small waves in clusters 3 and 4, the latter being more pronounced in January and February of 2023.

## 6 Applications

The evaluation of the supervised model and the classical outbreak detection method (EARS) in Table 2 shows distinct differences between regional aggregation level and model type. Across the board for all metrics and models, performance declines with finer regional granularity. This can be attributed to the characteristics of the time series which are more smooth at country level aggregation and more noisy at the county level (see Fig. 2(a–c)). Comparing the two models shows strong differences between the supervised RotationForest and the unsupervised EARS method. In terms of the machine learning metrics, more balanced results can be noted, while EARS leans strongly into specificity, producing very few false positives. Markedly, recall of the EARS algorithm is always around 0.16. It is relatively low, because of the nature of the output of the algorithm, as it produces prediction labels for single days, while the ground truth labels by our ensemble often span longer periods.

The timeliness metrics also differ a lot between both models, with the RotationForest always scoring much better. At the state and country level, the difference in days to detection (DTD) is around three days. At the county level, this is reduced to 1.19 days. There is a gap of 0.33 in the probability of detection within five days (POD_5_) between both models at the highest regional aggregation. The numbers converge to about 0.63 at the county level. In general it can be interpreted to say that the RotationForest is quicker in detecting relevant epidemiological events.

Please see the appendix for the results of other supervised models we tested (Table 4).

## 7 Discussion

In this work, we proposed a novel, ensemble-based approach to retrospectively label signals in time series. The ensemble approach was able to identify short-lived clusters of cases (outbreaks), as well as sustained, continuously increasing numbers of infections (waves) in the same time series. Our approach was to label COVID-19 time series that exhibits the aforementioned heterogeneous event-patterns. We have shown that our ensemble approach produced labels that behave consistent across time and space. On the country level, we show that our ensemble labels behave very similarly to manually curated expert labels as well as an independent wave detection framework. The number and length of produced labels had properties laying in between the single outbreak-focused labeling methods and the wave-focused methods.

We have provided an example in which the generated ensemble labels were used for a systematic evaluation of different outbreak detection methods. The evaluation was performed on a range of time series using several performance metrics with the ensemble labels as the ground truth. The possibility to compute labels for arbitrary time-series, instead of a costly manual labeling approach that is restricted to small sets of time-series, allows a more extensive evaluation on all levels, e.g. regional or demographic, of the surveillance data. Furthermore, such labels can be computed for datasets from surveillance systems that lack information on actual outbreak events or in cases where anomalous events in the data differ from known patterns seen in other diseases. Importantly, these labels are generated on real surveillance data, therefore also providing a more realistic benchmark for the comparison of outbreak detection methods.

We have demonstrated how to successfully use the generated ensemble labels to train supervised learning outbreak detection methods. Even standard models, that are not specialized on outbreak detection or time-series analysis, were able to outperform traditional unsupervised methods specialized for outbreak detection. This may give way to a new set of outbreak detection methods adapted to the properties of the real surveillance data and situation specific outbreak definition. Based on these findings, we are planning to deploy these methods in our outbreak detection tools that are used in practice by several regional and national public health institutes.

The proposed ensemble-approach can be easily extended through the addition of further labeling methods or adapted through the use of other parameter settings, voting schemes and post-processing steps to ensure minimum lengths of labels or maximum length of gaps between labels. Therefore, our approach can in theory be used to produce labels with a wide range of properties. Potential future research could focus on finding the optimal ensemble setting specifically for COVID-19 time series or time series of other diseases for which existing labeling methods may not be applicable.

Labels produced with this method can also be used as weak labels. As they are not produced by human experts, they cannot be relied on without further assessment by experts. However, they can be used to simplify human labeling by providing reasonable suggestions for labels that would only need to be adjusted. Such weak labels can also be used in a semi-supervised setting where expert labels (strong labels) are combined with weak labels to improve the performance of a supervised learning model compared to using only weak or strong labels.

The proposed process of retrospective labeling infectious disease time-series can be performed on many different surveillance datasets with very few requirements. Our open-sourced Python code allows public health researchers to easily apply this process on their own surveillance data. The resulting labels have many potential applications in public health in general and research on early warning methods in particular.

## CRediT authorship contribution statement

**Andreas Hicketier:** Writing – review & editing, Writing – original draft, Visualization, Validation, Software, Methodology, Investigation, Formal analysis, Data curation, Conceptualization. **Moritz Bach:** Writing – review & editing, Writing – original draft, Visualization, Validation, Software, Methodology, Investigation, Formal analysis, Data curation, Conceptualization. **Philip Oedi:** Writing – review & editing, Writing – original draft, Validation, Software, Methodology, Formal analysis, Data curation, Conceptualization. **Alexander Ullrich:** Writing – review & editing, Writing – original draft, Visualization, Validation, Supervision, Project administration, Methodology, Funding acquisition, Data curation, Conceptualization. **Auss Abbood:** Writing – review & editing, Writing – original draft, Validation, Supervision, Software, Project administration, Methodology, Funding acquisition, Data curation, Conceptualization.

## Declaration of competing interest

The authors declare that they have no known competing financial interests or personal relationships that could have appeared to influence the work reported in this paper.

## Figures and Tables

**Table 3:** Data format of the python package.

Column	Datatype	Input/Output	Description
value	integer	Input	Number of reported COVID-19 cases
target	string	Input	Reporting date (yyyy-mm-dd)
location	string	Input	The five-digit community identification code for counties, two-digit code for federal countries, and a 0 for the whole of Germany
label	$\left\{0,1\right\}$	Output	1 corresponds to a label, 0 to no label.

**Table 4:** Performance evaluation of other supervised machine learning models across different regions (DE: whole of Germany, BL: state level, LK: county level). Evaluation metrics used are precision, recall, specificity, days to detection (DTD), probability of detection (POD), and probability of detection within 5 days (POD_5_). Higher values are better for all metrics but DTD.

Region	Paradigm	Model	Prec.	Recall	Spec.	DTD	POD	POD_5_
DE	supervised	CNNClassifier	0.76	0.92	0.70	1.60	0.96	0.76
DE	supervised	InceptionTimeClassifier	0.70	0.83	0.58	3.40	0.85	0.76
DE	supervised	LSTMFCNClassifier	0.78	0.91	0.74	1.30	0.92	0.81
DE	supervised	TimeSeriesForestClassifier	0.76	0.79	0.76	1.77	0.92	0.76
BL	supervised	CNNClassifier	0.73	0.82	0.72	3.45	0.88	0.62
BL	supervised	InceptionTimeClassifier	0.70	0.64	0.76	6.75	0.79	0.55
BL	supervised	LSTMFCNClassifier	0.67	0.71	0.69	5.40	0.81	0.60
BL	supervised	TimeSeriesForestClassifier	0.70	0.74	0.73	3.26	0.87	0.66
LK	supervised	CNNClassifier	0.59	0.58	0.81	3.71	0.67	0.53
LK	supervised	InceptionTimeClassifier	0.54	0.55	0.72	3.77	0.68	0.55
LK	supervised	LSTMFCNClassifier	0.51	0.50	0.75	5.14	0.60	0.45
LK	supervised	TimeSeriesForestClassifier	0.52	0.48	0.76	4.40	0.64	0.45

**Fig. 4:**
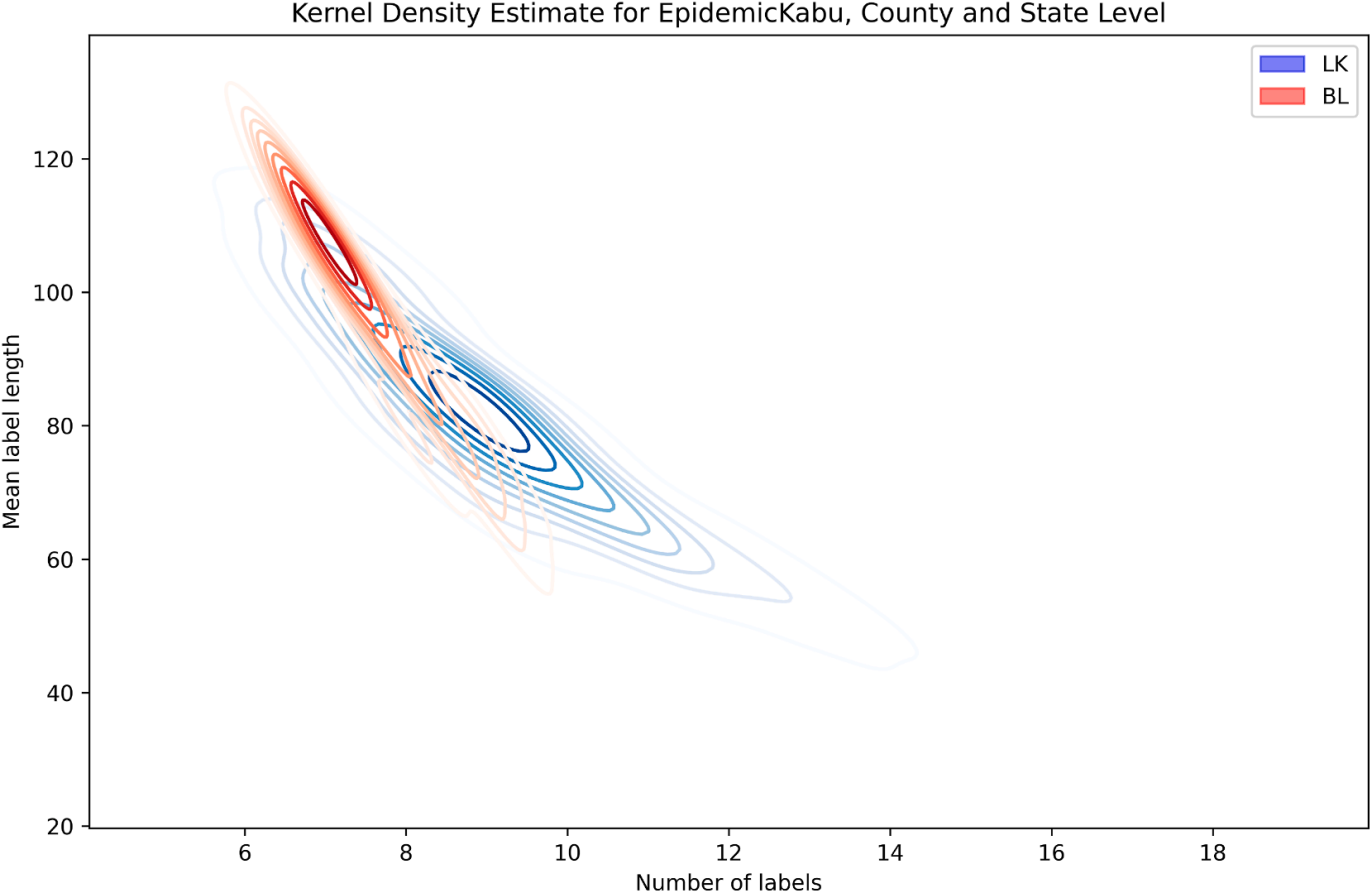
Kernel density estimate (KDE) plot of the EpidemicKabu method applied to our data for federal states (red) and counties (blue).

**Fig. 5:**
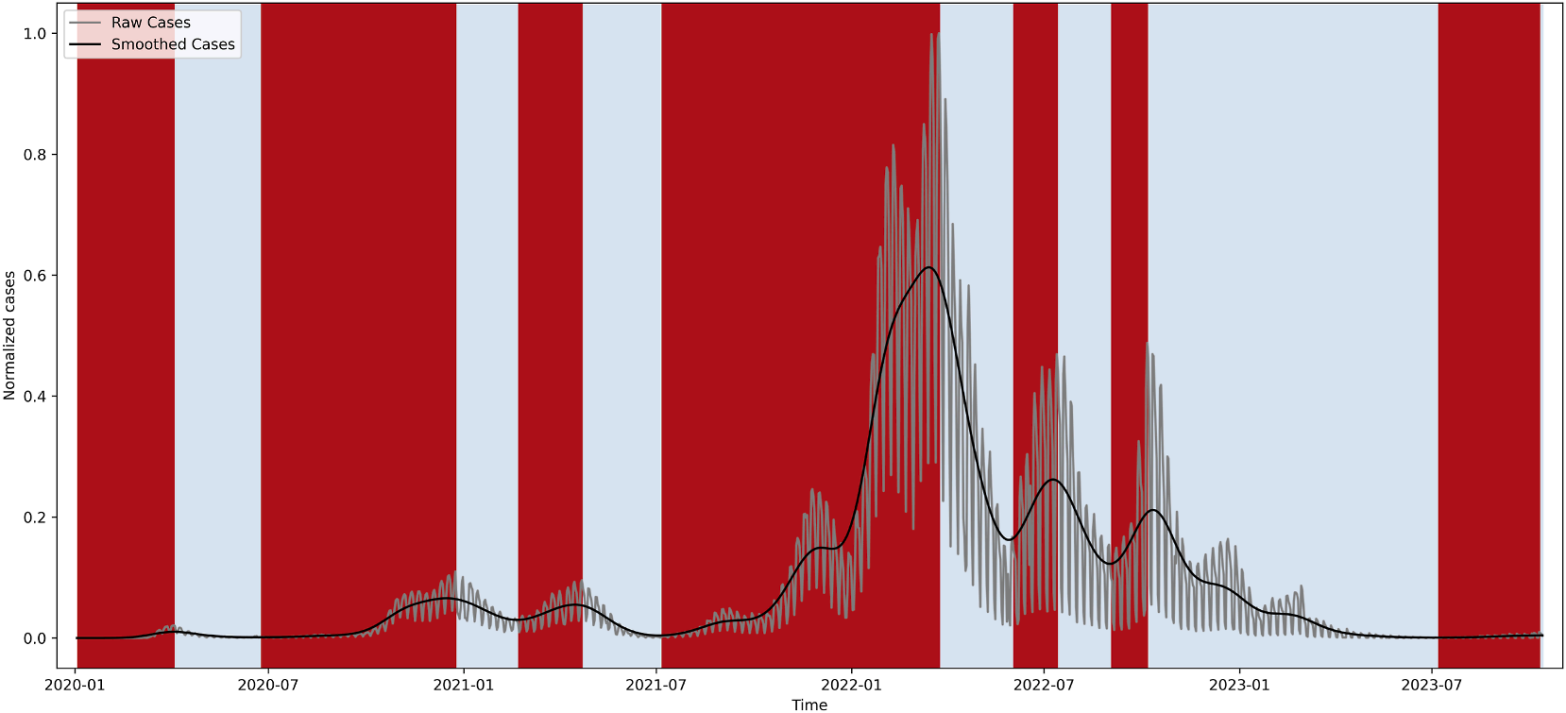
Raw and smoothed COVID-19 case counts in Germany and signal labels produced by EpidemicKabu on our data. Labeled time periods are indicated by red bands, otherwise the time series is gray.

**Fig. 6:**
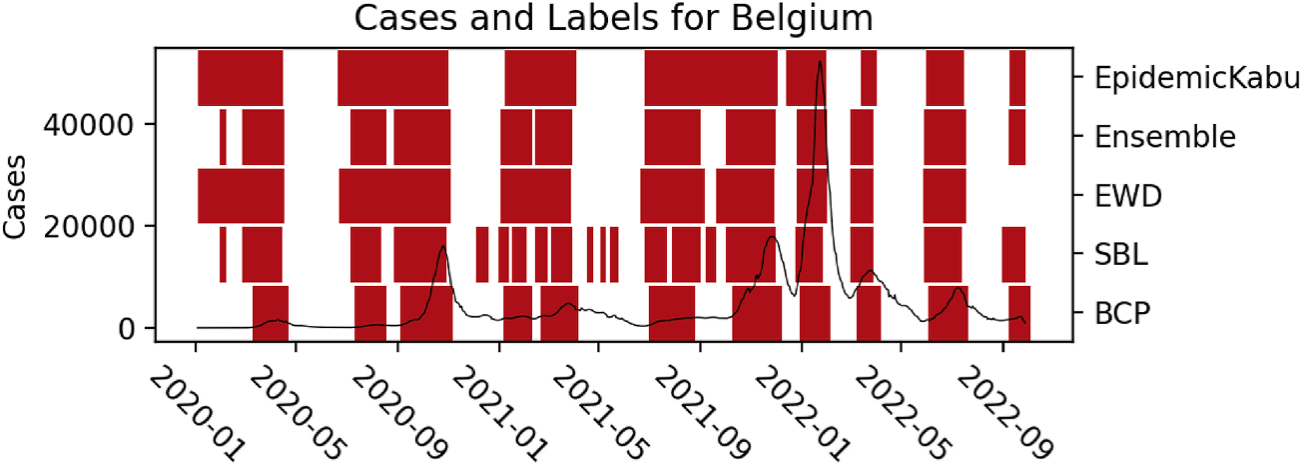
Data from EpidemicKabu.

**Fig. 7:**
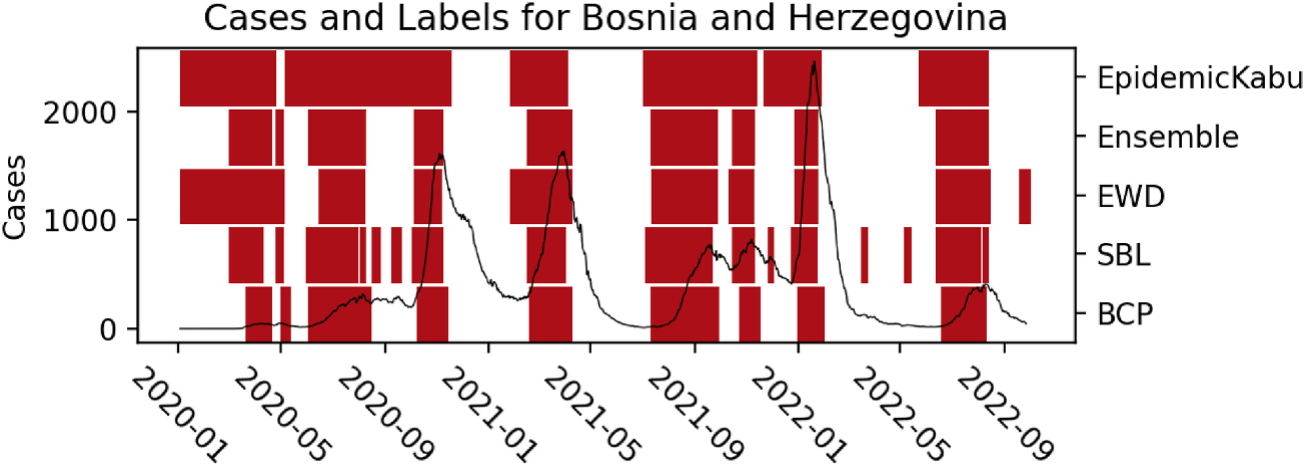
Data from EpidemicKabu.

**Fig. 8:**
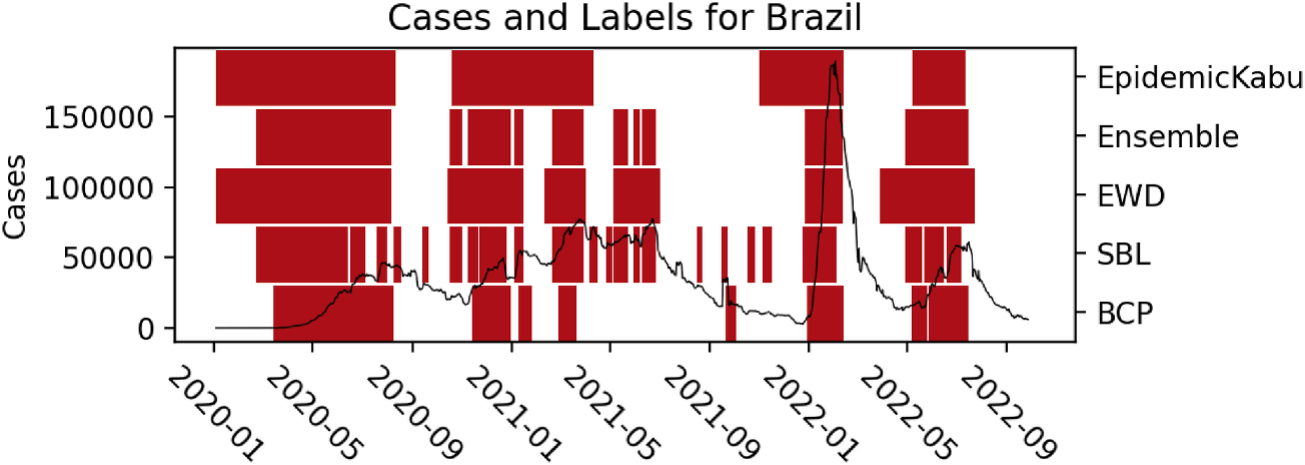
Data from EpidemicKabu.

**Fig. 9:**
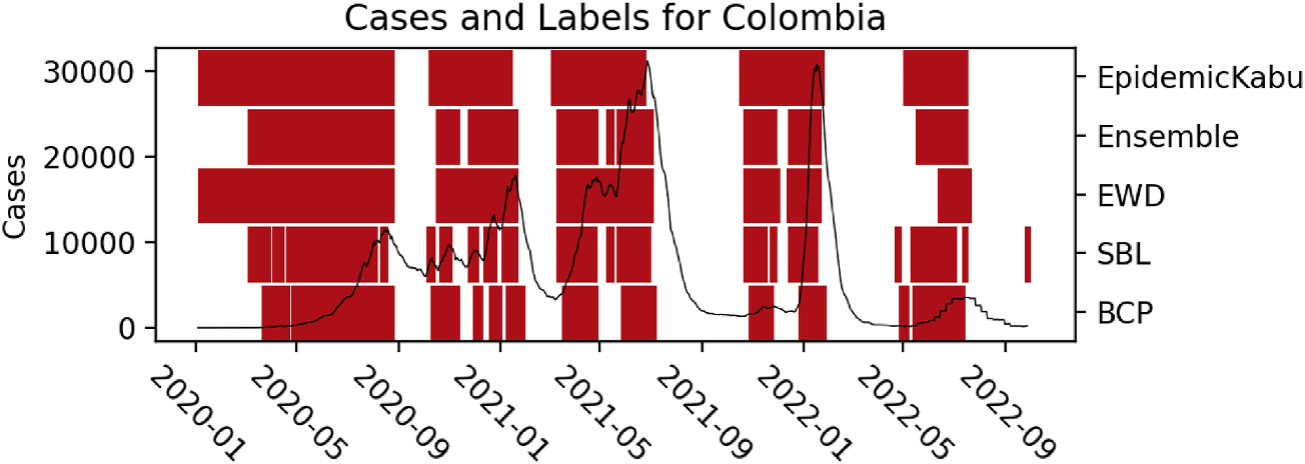
Data from EpidemicKabu.

**Fig. 10:**
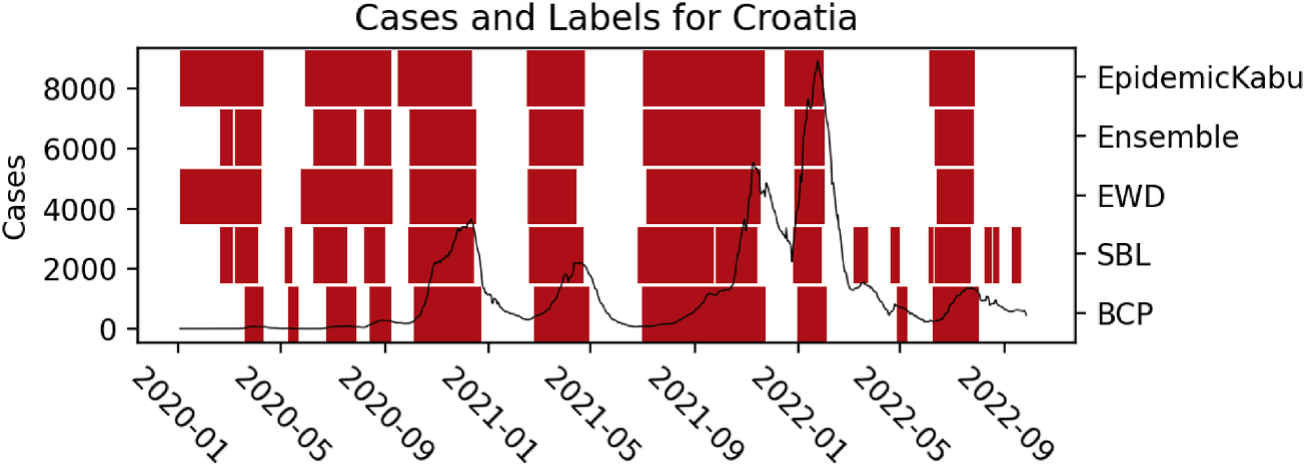
Data from EpidemicKabu.

**Fig. 11:**
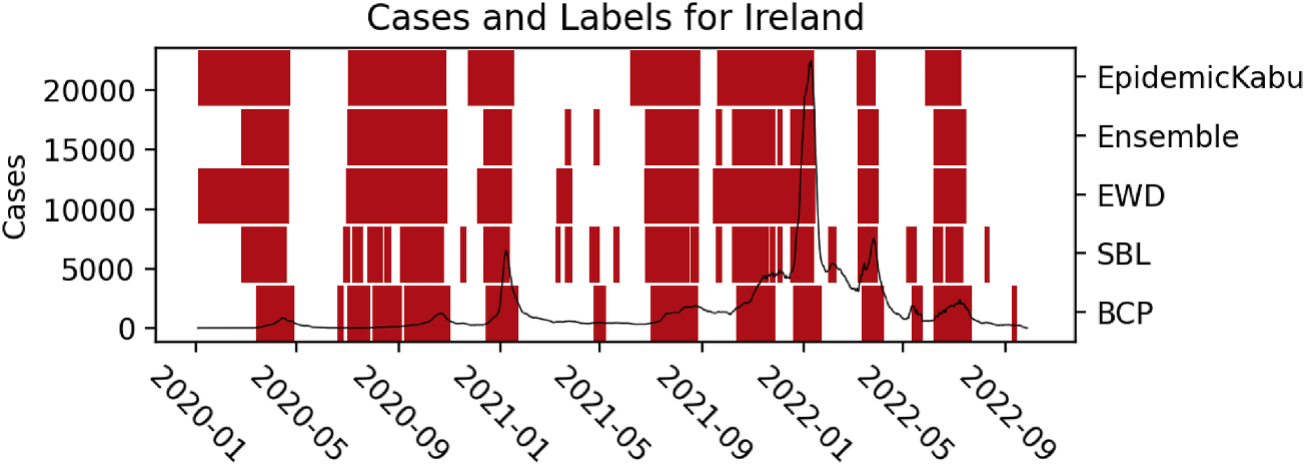
Data from EpidemicKabu.

**Fig. 12:**
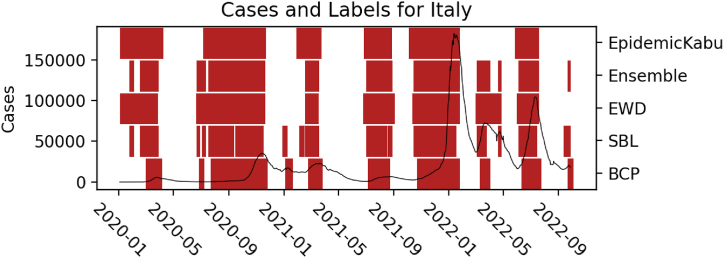
Data from EpidemicKabu.

**Fig. 13:**
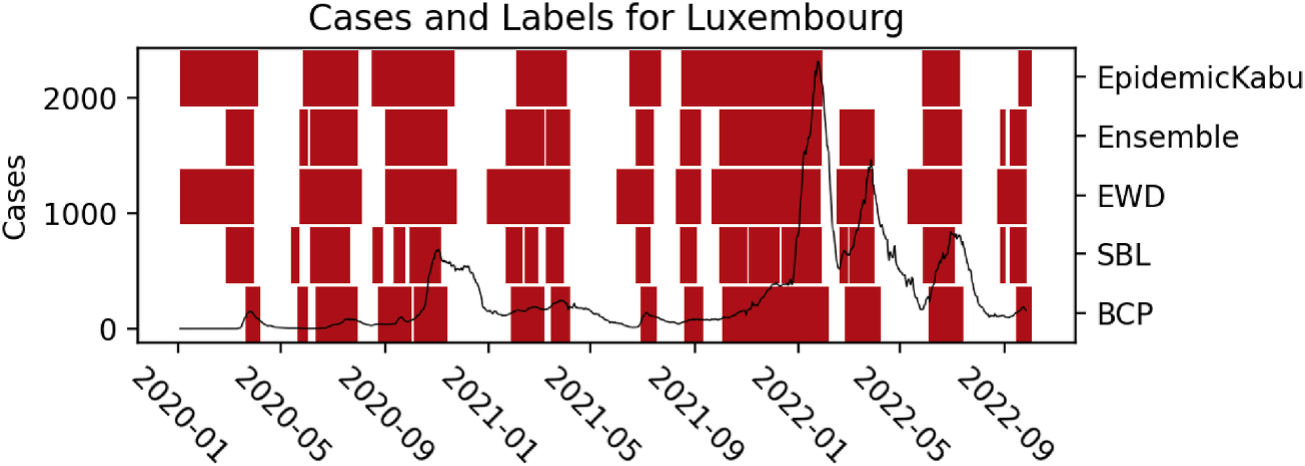
Data from EpidemicKabu.

**Fig. 14:**
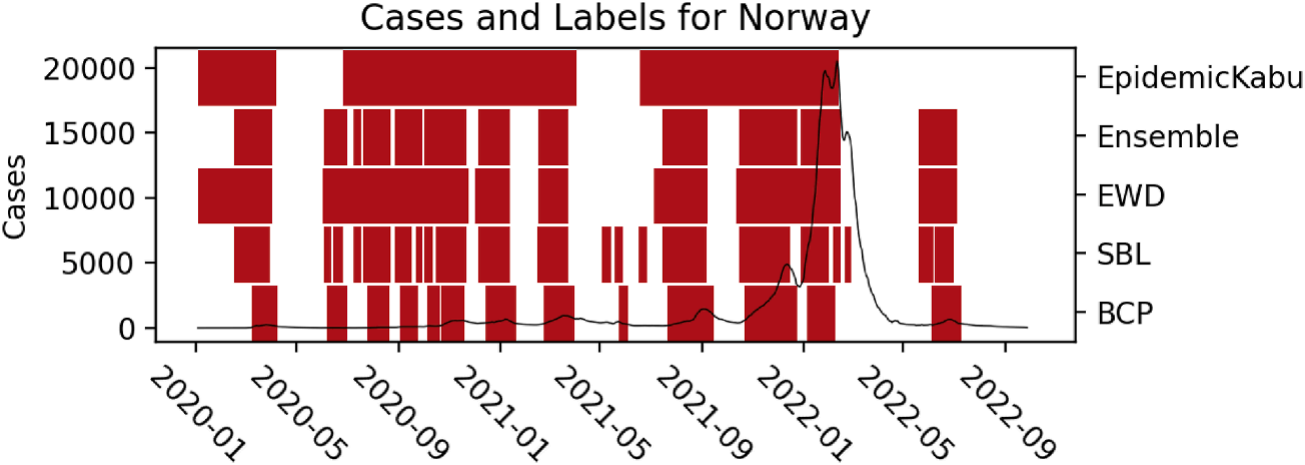
Data from EpidemicKabu.

**Fig. 15:**
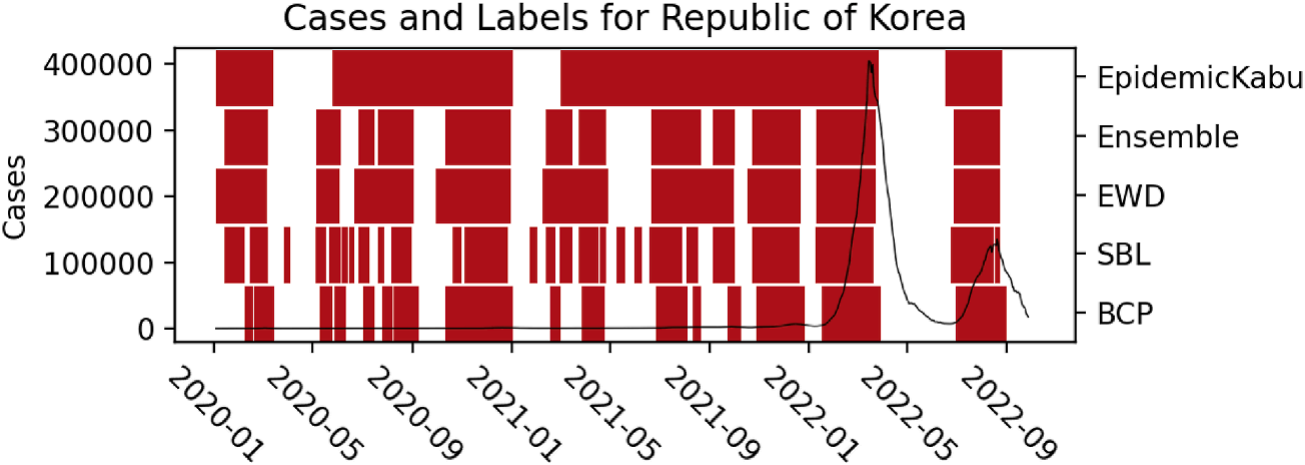
Data from EpidemicKabu.

**Fig. 16:**
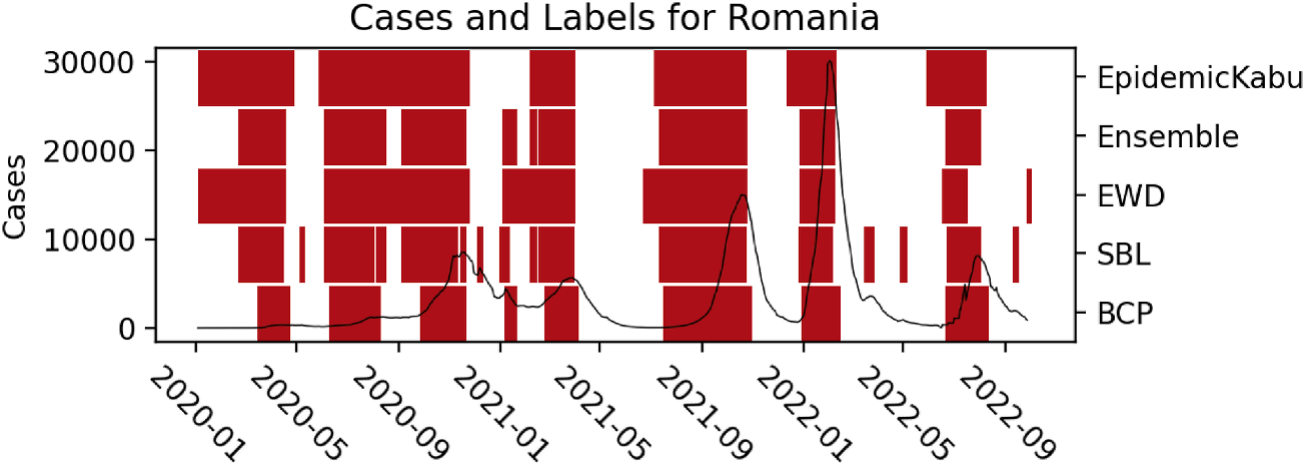
Data from EpidemicKabu.

**Fig. 17:**
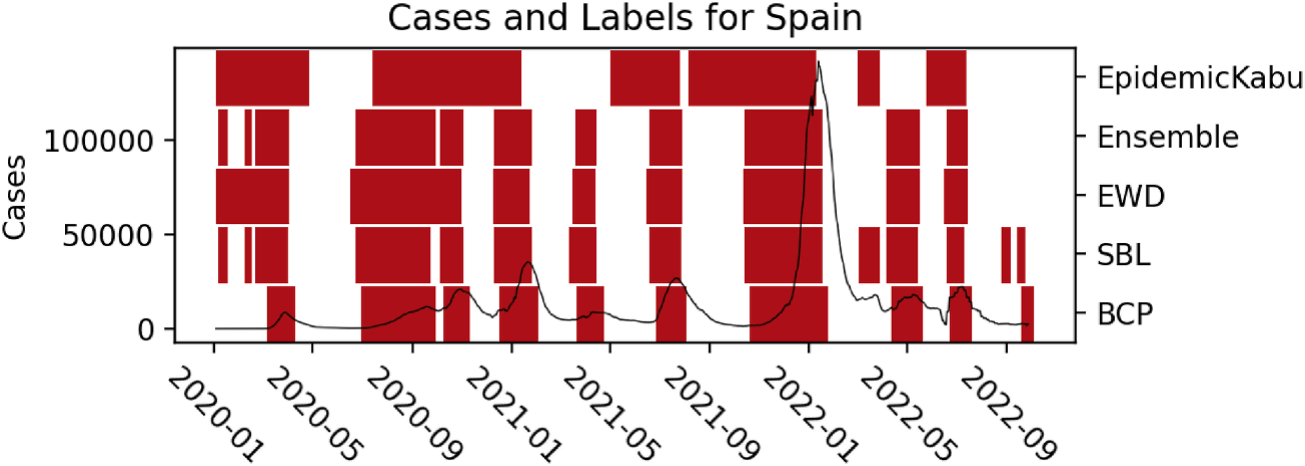
Data from EpidemicKabu.

**Fig. 18:**
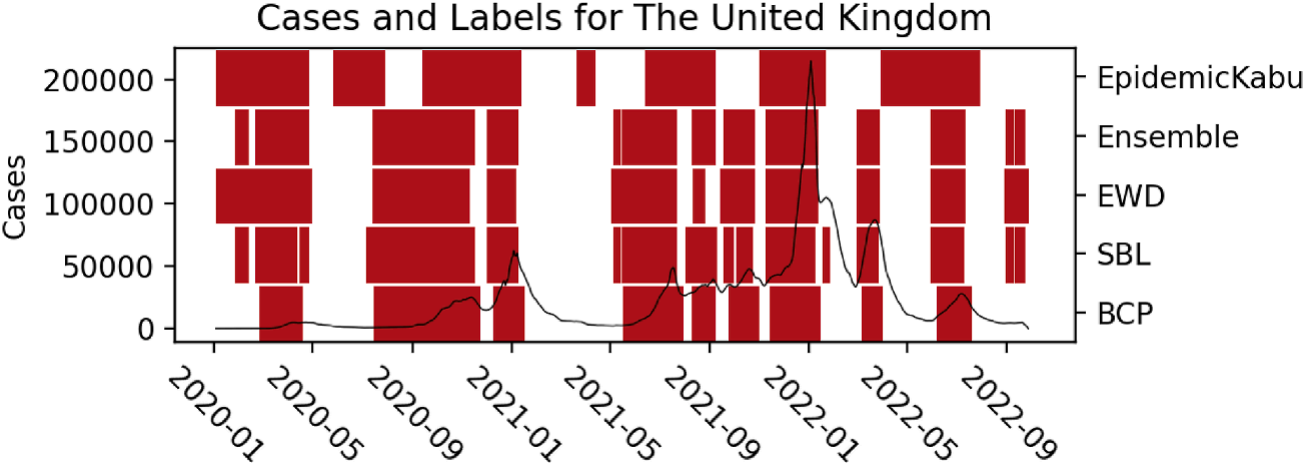
Data from EpidemicKabu.

**Fig. 19:**
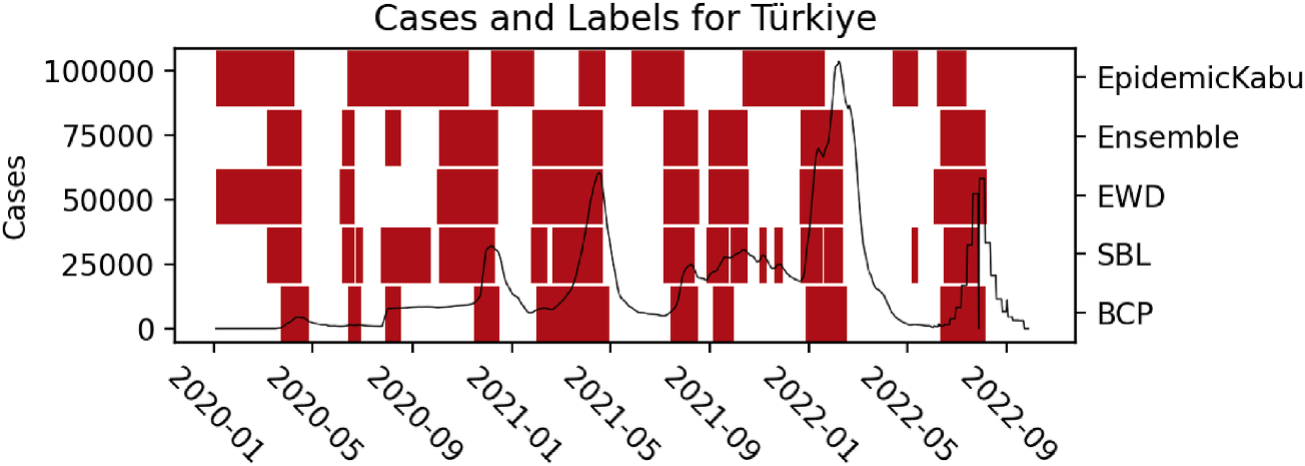
Data from EpidemicKabu.

**Fig. 20:**
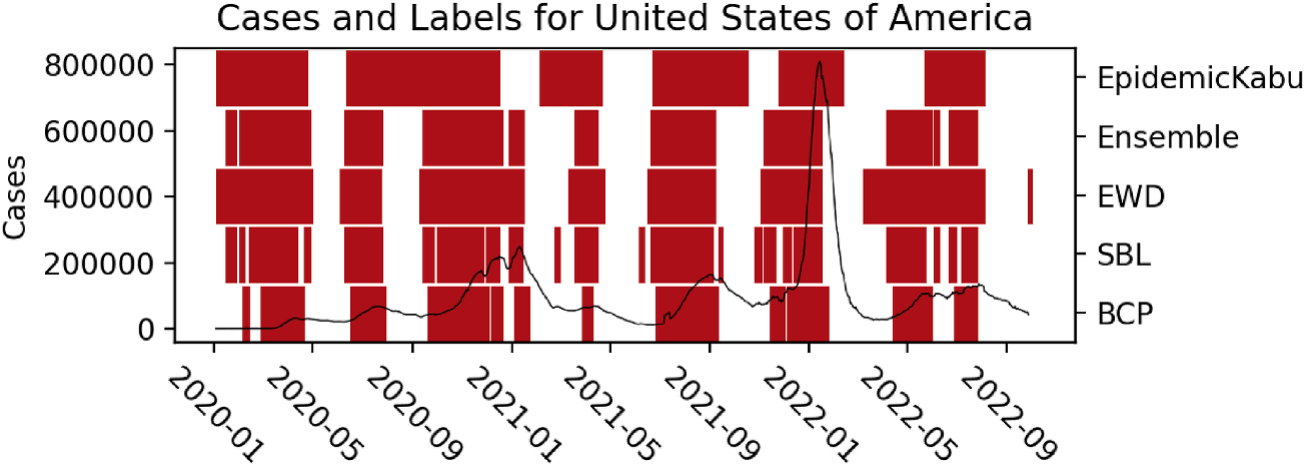
Data from EpidemicKabu.

## Appendix

### 11.1. Data

Table 3 shows the data format that all time series in this paper follow.

### 11.2. Full supervised model results

Table 4 shows the full results of all tested models not reported in section 3.3. Model performance was mostly dependent on regional level of the time series while differences between models are not consistent.

### 11.3. Comparison with another wave detection method

We additionally compare our ensemble-labeling approach with the recently proposed wave detection method EpidemicKabu (Galvis et al., 2024). To this end, we first applied EpidemicKabu to the German COVID-19 7-day-incidence time series used in this study (country, federal state, and county level). In a second step, we applied our ensemble method to the COVID-19 case time series from 15 countries that were analysed in the original EpidemicKabu publication, which the authors provide in their code repository https://github.com/LinaMRuizG/EpidemicKabuLibrary.

EpidemicKabu normalizes the case counts of each time series by their respective global maximum and then smooths the time series with a Gaussian kernel. For the German data, we estimate the kernel radius automatically using the procedure proposed by the authors, which derives the radius from the standard deviation of the normalized case counts. Regarding the data of the other 15 countries, we use the country-specific kernel radii provided in the configuration file of the EpidemicKabu code repository. After smoothing, the algorithm computes the discrete first derivative of the time series, smooths it again, and defines candidate wave boundaries at zero-crossings where the derivative changes sign from negative to positive. These candidates are filtered using the second derivative and the default threshold. This combination of Gaussian smoothing and derivative-based zero-crossing criteria suppresses short-lived fluctuations and places boundaries only at pronounced local minima of the smoothed curve, so that the resulting segments correspond to coherent epidemic waves rather than noise-driven artifacts. Similar to the EWD, EpidemicKabu marks the whole period from beginning of a wave to the beginning of the next wave as “wave”. We are only interested in the increasing part. For a fair comparison with our methods, we define the end point of each EpidemicKabu wave as the date of its maximum, which is consistent with how we use the EWD.

Compared to our ensemble method, EpidemicKabu produces fewer but substantially longer labels (Fig. 2 (d) and Fig. 4). For the ensemble, Fig. 2 (d) shows a clear separation between federal states and counties: county time series contain many short labeled periods, whereas federal states exhibit fewer but much longer labels. For EpidemicKabu this spatial pattern is much less pronounced; in Fig. 4 the KDEs for states and counties largely overlap, indicating similar numbers and lengths of labels across aggregation levels.

Among the methods in our ensemble, the Epidemiological Wave Detection method (EWD; Fig. 2) is by construction most similar to EpidemicKabu, and the resulting labels reflect this (Fig. 2(e)). Nevertheless, EpidemicKabu still tends to detect fewer and longer waves compared to EWD (Figs. 2(a) and 5). The same qualitative pattern emerges for both data sets (Figs. 6–20).

While the methods in our ensemble operate on mildly smoothed data such as the 7-day incidence, EpidemicKabu requires stronger Gaussian smoothing of the raw daily case counts in order to obtain stable discrete derivatives. This stronger smoothing suppresses shorter or more moderate fluctuations in the incidence curves and therefore leads to fewer, longer labels and less nuanced wave dynamics. Despite this coarser representation, the wave onsets and offsets identified by EpidemicKabu are largely consistent with those obtained from our ensemble, indicating broad agreement on the underlying wave structure. This robustness across conceptually different approaches both highlights the practical usefulness of EpidemicKabu and provides additional validation for our ensemble-based method.
